# Interferons as Therapy for Viral and Neoplastic Diseases: From Panacea to Pariah to Paragon

**DOI:** 10.3390/ph2030206

**Published:** 2009-12-15

**Authors:** Robert M. Friedman, Sara Contente

**Affiliations:** Department of Pathology, F. Edward Hébert School of Medicine, USUHS/4301 Jones Bridge Rd, Bethesda, MD 20814, USA; Email: scontente@usuhs.mil (S.C.)

**Keywords:** interferon, clinical applications, cancer, infectious diseases, multiple sclerosis

## Abstract

For more than 20 years after the excitement engendered by their discovery in 1957 as antiviral agents, there were no significant clinical uses of interferons; however, following their cloning they have been employed as effective treatment for several viral, autoimmune, and neoplastic diseases.

## 1. Introduction

After the first description of interferons in 1957 [1], their antiviral activity attracted wide interest, and there was some expectation that they would be developed clinically as agents to treat viral infections. A number of problems arose, however, that delayed their clinical use. The first of these was the finding that the interferons are species specific in their biological activity [2]. With a few rare exceptions, only human or primate interferons are active in humans, so the single source of interferons for human use in the 1960s and 70s was primate cells, and the supply of such cells was quite limited. Furthermore, when it was discovered that interferons possessed then unprecedented biological activity, it became evident that existing stocks of interferons with very significant antiviral activity actually were quite impure and actually contained very little interferon. Because of this lack of pure, or for that matter even moderately clean interferon, it was impossible to accept any biological activity of an interferon preparation, other than antiviral activity, as being due to its interferon content. 

In spite of these problems, there were early clinical trials of the antiviral activity of what interferon supplies were then available. These studies involved, for the most part, the ability of an interferon produced by simian cells to inhibit the development of vaccinia virus lesions in human skin, or respiratory infections following exposure of volunteers to common cold viruses [3,4]. The results of these studies were unimpressive, almost certainly because of the small quantities of impure interferon employed in these studies; consequently, for years studies on interferons were limited to experiments in tissue cultures, and to attempts to produce and purify sufficient quantities of interferon from human white blood cells to carry out significant clinical studies.

## 2. Clinical Uses of interferons

Interest in the possible clinical studies employing interferons picked up in the mid-1970s when sufficient quantities of human purified (but not pure) IFN-α, obtained from the white blood cells (buffy coat) of donated blood by Cantell’s group, became available [5] for a small number of clinical experiments, many of which had promising, if not highly significant results in studies on the prevention of common colds [6] and the treatment of several herpes virus infections, such as herpes keratoconjunctivitis and the varicella-zoster infections, shingles and chickenpox [7,8]. The discovery that interferon in tissue culture inhibited chronic infections with mouse leukemia viruses [9] prompted additional studies employing interferon as therapy for chronic hepatitis B virus (HBV) infections. These had very promising results [10]. Also encouraging were the experiments of Jacobs et al who reported that multiple sclerosis (MS) patients, treated with partially purified human IFN-β administered by lumbar puncture had a significant decrease in the frequency of the periodic exacerbations characteristic of that disease [11].

Interest in the possible clinical use of interferons for treatment of malignancies was greatly stimulated by promising results of limited clinical trials, most of which employed interferon supplied by Cantell’s laboratory, for treatment of renal cancers [12], malignant melanomas [13], lymphomas, and leukemias [14]. Such studies followed a number of reports that type I interferons were effective in treating cancers in mice [15]. Interest in interferons as a treatment of cancers was further stimulated by a report from Sweden that osteosarcomas responded favorably to treatment with IFN-α, a study that subsequently was shown to be flawed [16]. These preliminary studies had both positive and negative consequences: That interferons might be potential anticancer drugs led to widespread, unwarranted expectations of them being a general cure for cancer; on the positive side, however, interest in finding better sources for a potential wonder drug, led directly to the cloning of genes for IFN-α [17], and later, for the genes for IFNs -β and -γ [18,19]. This in turn led to the production of quantities of pure interferon sufficient to carry out a significant number of clinical trials with meaningful results. Such studies have clarified what the role of interferons might be in the treatment of a number human diseases, so that recombinant IFN-αs are widely employed with some success in the treatment of chronic HBV and hepatitis C virus (HVC) infections and some forms of cancer. IFN-β treatment is regularly used to limit exacerbations of MS [20]. IFN-γ has been approved for clinical use only in a rare congenital disorder, chronic granulomatous disease, for which it is effective in preventing bacterial infections. A recent clinical trial of IFN-γ for the treatment idiopathic pulmonary fibrosis patients did not improve their survival [21]. Current clinical trials are underway employing, in the treatment of chronic HBV and HVC infections, interferon lambda (IFN-λ), which is biologically similar to IFN-α and -β, but employs a different membrane receptor [22,23]. Phase I trials were successfully completed in October, 2009, and Phase 2 trials have been initiated.

### 2.1. Use of IFN-α in chronic HCV infection

By far the best understood clinical application of interferons is against chronic HCV infections, for which IFN-α has been an approved treatment since 1991 [24]. HCV is a widespread infection spread by contaminated blood products or by drug injection. Although modern blood bank technology has almost eliminated the former, the latter remains a major, world-wide problem. There are millions of HCV-infected patients. The progress of HCV infections is insidious, often not being clinically manifest for two or three decades after initial infection with the virus. Chronic HCV infection may cause serious hepatic malfunction eventually resulting in cirrhosis of the liver and in life-threatening esophageal varices. A significant number of HCV patients eventually develop hepatocellular cancers (hepatomas). Chronic infections with hepatitis B (HBV) and HCV are a significant cause of death in patients with AIDS [25].

HCV is a small ribovirus with six genotypes, of which genotype 1, unfortunately the most common infection in North America, is relatively insensitive to IFN-α. It appears possible to predict the response of a patient to infection with a genotype 1 HCV isolate by use of structural analysis of the infecting virus [26]. The core protein of genotype 1 HCV induces cellular proliferation and transformation, and so is associated with advanced hepatic cirrhosis and hepatocellular transformation [27]. The resistance to IFN resides in a nonstructural viral protein, a serine protease that inactivates the signal leading to interferon production, thus facilitating the development of chronic infections [28]. Current treatments for chronic HCV infections have several limitations, as they result in rates of sustained virus responses that were lower in black and Latino patients than in non-Latino whites [29,30]. Long-term, IFN-based treatment did not halt the progression of chronic HCV infections in patients, not responding to initial treatment [31]. IFN-α is also useful in the treatment of cryoglobulinemia, a complication of chronic HCV infections [32]

In order to augment the effectiveness of IFN-α employed in the treatment of HCV, two alterations in the protocol for the infection were initiated: Ribavirin, an oral nucleoside analogue that inhibits the growth of some RNA viruses, was added to the regimen [33]; and, the IFN-α was conjugated to polyethylene glycol (peginterferon), thus decreasing its renal clearance and significantly increasing its half-life from about 5 h to almost 90 h, and so decreasing the number of injections required in a treatment course [34]. With the combined ribavirin/peginterferon treatment more than 75% of nongenotype 1 HCV patients maintain a sustained anti-HCV response, while up to 50% of the patients infected with the genotype 1 HCV responded to this combined treatment, and in those patients responding to peginterferon/ribavirin therapy, virus-induced liver damage failed to progress, with some degree of healing taking place [34]. IFN-based treatment was associated with improved survival and reduced the risk of hepatocellular cancer. Long-term follow up indicated that once a particular HCV-infected patient attains a sustained response to peginterferon/ribavirin therapy, defined as undetectable levels of HCV RNA in the serum for six months, the risk for virologic relapse is very low [35]. In one clinical study, low doses of peginterferon and ribavirin were as effective as higher dose levels [31]. Unfortunately, the prolonged peginterferon therapy necessary to control chronic HCV or HBV infections was often associated with serious side-effects such fever, depression, and muscle pain, as discussed below [25]. In patients who did not respond to standard peginterferon/ribavirin therapy, substitution of the consensus interferon, alfacon-1, plus ribavirin proved effective in some patients [36]. Currently, new forms of therapy to augment treatment with ribavirin/peginterferon are under development, including inhibitors of the HCV protease, helicase, or polymerase and IFN-α conjugated to albumin [37]. Combinations of such agents with the currently employed therapies may provide effective treatment for a much larger percentage of HCV patients then are currently responding to anti-HCV treatment [34].

### 2.2. Use of interferons in chronic HBV infection and tumorigenic viral infections

Chronic HBV infection is the other major antiviral application of interferons [38]. HBV is a hepatotropic DNA virus. Like HCV, HBV infection is associated with contaminated blood or blood products, but unlike HCV is also a sexually transmitted disease. An effective vaccine for HBV has greatly decreased the incidence of this disease, but a large number of chronically infected patients remain. At present, chronic infections with HBV are treated with a combination of peginterferon and one of several nucleotide analogues that inhibit the replication of the HBV genome such as lamivudine, adefovir, entecavir, or tenofovir. New, more effective anti-HBV nucleotide analogues are under development. With peginterferon/lamivudine therapy for one year, patients have significant anti-HBV responses, including decreases in serum HBV DNA, HBV antigen seroconversion, normalization of serum transaminase, and histologic improvement in inflammatory liver lesions [39,40]. Lymphoblastoid interferon has also proven effective in the treatment of HCV infections [41]

IFN-α is also employed in the treatment of infection caused by human herpes virus-8 (HHV-8), the etiological agent in Kaposi’s sarcoma (KS), formerly a rare form of cancer that has become prominent because of its association with AIDS [42]. KS is a multifocal tumor of the vascular endothelium, the most frequent manifestation of which is the development of multiple purple skin nodules. HHV-8 propagates in AIDS patients due to the suppression of the immune response associated with their disease. In addition, HHV-8 produces a protein that directly inhibits interferon induction. Since KS is caused by a virus, it was logical to attempt to treat the disease with IFN-α. This treatment can be local, directly applied to skin lesions, or systemic. The success rate approaches 60% when the therapy with IFN-α is combined with effective antiretroviral treatment for AIDS [43]. 

A few other human tumors which are caused by infection with members of the human papillomatosis virus (HPV) group have been treated with interferons. These include recurrent respiratory papillomatosis [44] and genital warts [45]. Juvenile laryngeal papillomatosis, which can be a life-threatening disease, has been treated with IFN-α for over 20 years [46]. Such treatment is effective in infections with HPV types that have a low potential for inducing malignant transformation, but survival of patients is poor in infections with types with a high rate of cancer induction. Genital warts, too, are induced by HPV infections, and may be treated by interferons. However, a number of less expensive forms of therapy are also available for this disease, so that interferons are no longer recommended as its primary mode of treatment [45].

### 2.3. Use of interferons in treatment of neoplasms

Because of their cell differentiation and growth regulatory properties, interferons have been used in attempts to treat neoplasms. Several forms of hematological cancers and solid tumors have been treated with interferons with some success. However, as little as is understood of the mechanism of antiviral actions of interferons, there is even less understood about their effectiveness, such as it is, in cancer therapy. Even before recombinant interferons were available, remissions were reported with partially purified IFN-α in hairy-cell leukemia [47], a slowly progressive, rare form of B-cell leukemia. Some success was also reported with treatment of chronic myelogenous leukemia (CML), a more common B-cell form of leukemia [48]. In both of these leukemias, however, more effective forms of therapy have been devised through our rapidly developing understanding of the molecular mechanisms involved in the genesis and progression of some forms of cancer, so that interferons are no longer employed as first-line therapies in these diseases [49]; however, a recent report suggests that IFN-α causes hematopoietic stem cells in CML to enter the cell cycle, thus inducing an augmented susceptibility to their destruction by imatinib, the most effective chemotherapeutic agent currently available for treating this form of leukemia [50]. IFN-α has also been employed with moderate success in the treatment both basal and squamous cell skin carcinomas [51,52]. Adjuvant therapy with IFN-α of patients with hepatocellular carcinomas is reported to be associated with prolonged survival in Chinese males (90% chronically infected with HBV), with reduced expression levels of the microRNA mIR-26 [53]

There are two relatively immunogenic forms of cancer for which interferons are still commonly employed as therapy, mainly because no alternative treatment has been found. About 15% of metastatic renal-cell carcinomas respond to treatment with IFN-α alone. However, when employed in the treatment of this form of cancer, IFN-α is used in conjunction with other forms of biological response modifiers such as interleukin-2 or the vascular endothelial growth factor inhibitor bevacizumab [54]; however, as in the case of chronic myelogenous and hairy cell leukemias, more effective forms of therapy for renal-cell cancers are now available [55]. The other major cancer for which interferon is employed as adjuvant therapy is cutaneous melanoma that has metastasized to local lymph nodes [56]. Since malignant melanomas are probably the most capricious form of cancer, it is difficult to evaluate the effectiveness of any therapy for them. A number of reports, however, do show improved survival, a decreased rate of relapse, and an improved quality life in melanoma patients treated with high dosages of IFN-α. Because of the toxicity associated with prolonged, high doses of interferon, a number of European studies of lower doses of interferon were undertaken. However, none of the low dosage regimens has so far been associated with a significant recurrence-free patient survival [56].

The anti-angiogenic property of interferons has been employed for the treatment of very large, life-threatening hemangiomas (benign tumors of blood vessels) in infants. These hemangiomas usually respond to corticosteroid therapy. However, where this treatment has been ineffective, IFN-α therapy for several weeks or months has been life-saving in cases where steroids have failed [57]. Peginterferon-α2a has been successfully employed in the treatment of polycythemia vera [58] and chronic immune thrombocytopenic purpura, in the latter case when employed in conjunction with the megakaryocyte-stimulating agent romiplostim [59].

### 2.4. Use of interferon in autoimmune disease and multiple sclerosis

Oral administration of IFN-α has been employed in animal experiments and in pilot clinical studies to treat autoimmune diseases such as type 1-diabetes, Sjogren’s syndrome, and rheumatoid arthritis with some reported success in decreasing immune responses to antigens responsible for these disorders. The mechanisms involved in these reported favorable clinical responses would appear to be different from those reported in the antiviral and tumor inhibitory activities of IFN-α [60].

Untreated multiple sclerosis (MS) is a relentlessly progressive neurodegenerative disease, which usually develops as a series of remissions and relapses as a result of the demyelization of nerves. Treatment of MS with lFN-β was initiated based on their immunomodulatory properties [61]. Because of the unpredictable course of MS, judging the effectiveness of any form of therapy for this disease has been difficult; however, well-controlled studies have demonstrated that intramuscular treatment with lFN-β results in a reduction in the annual rate of relapses of MS. Initiation of lFN-β treatment with the first instance of demyelization appears to be a justified therapeutic intervention [20]. Addition of natalizumab, a recombinant monoclonal antibody to an integrin, augments the ability of lFN-β to decrease the rate of MS progression. Employed together, natalizumab and IFN-β treatment resulted in about a 50% reduction in the rate of MS relapses, when compared with that observed with IFN-β alone [62]. Lymphoblastoid interferon has also proven useful in the treatment of chronic progressive MS [63].

## 3. Conclusions

From the time of their first uses in patients, IFNs has been observed to have a number of characteristic toxic effects. These include influenza-like symptoms such as fatigue, fever, and myalgias. Indeed, it is thought that these symptoms, characteristic of many acute viral infections, are due to the stimulation of IFN production by the infecting agent, and they may be in some patients relieved by treatment with aspirin. Other psychiatric changes such as depression, anxiety, and irritability may require treatment with psychoactive pharmaceuticals. More severe toxicities such as cytopenias and autoimmune disorders have also been reported in patients treated with IFNs [64].

Interferons were among the first proteins to be cloned, and so research on them has contributed significantly to the rapid development of molecular biology. They are now considered to be members of the cytokine and growth factor class of biologically active agents. Because research on interferons had been initiated well before the other members of the cytokine and growth factor class were discovered, interferons were seminal in the development of research and clinical studies on this group. There is very little understanding of the mechanisms of almost all of the clinical applications of the interferons, so further insight into them may well lead to the development of pharmacologically active agents that are more specific and potent.
